# Upper- vs. Lower-Body Exercise Performance in Female and Male Cross-Country Skiers

**DOI:** 10.3389/fspor.2021.762794

**Published:** 2021-12-21

**Authors:** Linda Marie Hansen, Øyvind Sandbakk, Gertjan Ettema, Julia Kathrin Baumgart

**Affiliations:** Department of Neuromedicine and Movement Science, Centre for Elite Sports Research, Norwegian University of Science and Technology, Trondheim, Norway

**Keywords:** oxygen uptake, heart rate, blood lactate, running, XC skiing, XC skiers, double poling, upper-body poling

## Abstract

**Purpose:** To investigate the interaction between exercise modality (i.e., upper- and lower-body exercise) and sex in physiological responses and power output (PO) across the entire intensity spectrum (i.e., from low to maximal intensity).

**Methods:** Ten male and 10 female cross-country (XC) skiers performed a stepwise incremental test to exhaustion consisting of 5 min stages with increasing workload employing upper-body poling (UP) and running (RUN) on two separate days. Mixed measures ANOVA were performed to investigate the interactions between exercise modalities (i.e., UP and RUN) and sex in physiological responses and PO across the entire exercise intensity spectrum.

**Results:** The difference between UP and RUN (Δ_UP−RUN_), was not different in the female compared with the male XC skiers for peak oxygen uptake (18 ± 6 vs. 18 ± 6 mL·kg^−1^·min^−1^, *p* = 0.843) and peak PO (84 ± 18 vs. 91 ± 22 W, *p* = 0.207). At most given blood lactate and rating of perceived exertion values, Δ_UP−RUN_ was larger in the male compared with the female skiers for oxygen uptake and PO, but these differences disappeared when the responses were expressed as % of the modality-specific peak.

**Conclusion:** Modality-differences (i.e., Δ_UP−RUN_) in peak physiological responses and PO did not differ between the female and male XC skiers. This indicates that increased focus on upper-body strength and endurance training in female skiers in recent years may have closed the gap between upper- and lower-body endurance capacity compared with male XC skiers. In addition, no sex-related considerations need to be made when using relative physiological responses for intensity regulation within a specific exercise modality.

## Introduction

During training and competitions, cross-country (XC) skiers employ various sub-techniques that differ considerably in the amount of upper- and lower-body propulsion. For example, the double-poling sub-technique requires a large contribution from the upper body, whereas the diagonal sub-technique relies more on the lower body (Sandbakk et al., 2014). Accordingly, XC skiers train using various upper- and lower-body exercise modalities, with most of the training being conducted as low-intensity endurance training interspersed by key sessions at moderate and high intensity (Sandbakk and Holmberg, 2017). During endurance training, physiological responses such as blood lactate concentration (BLa) and percentage of peak heart rate (%HR_peak_), as well as perceptual parameters such as rating of perceived exertion (RPE) are used to determine exercise intensity. While some of these parameters differed between upper- and lower-body exercise (Sawka et al., 1982; Undebakke et al., 2019), and between female and male endurance athletes, the interactions between exercise modality and sex have not yet been investigated across the entire exercise intensity spectrum, from low to maximal intensity.

Modality-specific peak oxygen uptake ($\dot{\text{V}}$O_2peak_) and peak heart rate (HR_peak_) are clearly lower during upper- compared with lower-body exercise (Sawka et al., 1982; Undebakke et al., 2019). This indicates that the cardiovascular system is taxed less when exercising with the smaller active muscle mass of the upper body (Miles et al., 1989). Furthermore, male skiers have a higher peak power output (PO_peak_) and $\dot{\text{V}}$O_2peak_ compared with female skiers during both upper- and lower-body exercise (Janssen et al., 2000; Sandbakk et al., 2012; Hegge et al., 2016). Until recently the differences between exercise modalities (hereafter referred to as differences between upper-body exercise and running, Δ_UP−RUN_) were smaller in male athletes (Maldonado-Martin et al., 2004; Sandbakk et al., 2014; Sandbakk and Holmberg, 2017), which was related to the relatively larger muscle mass in the upper body of men (Janssen et al., 2000; Hegge et al., 2016). The authors explained these findings with a larger focus on endurance and strength training of the upper body among male XC skiers (Hegge et al., 2016). Accordingly, it has been argued that incorporating more upper-body specific training among female XC skiers could potentially result in an improved (upper-body) peak performance (Vandbakk et al., 2017) and reduced Δ_UP−RUN_ differences for peak physiological responses and PO. In this context, unpublished data from our group also indicate higher volumes of upper-body-specific training in female XC skiers in recent years.

A possible improvement in the peak physiological responses in female XC skiers during upper-body exercise may also be reflected in the Δ_UP−RUN_ differences between sexes at submaximal intensity. To this date, studies have focused on either the comparison of submaximal responses between upper- to lower-body exercise without considering sex, or the comparison between sexes within upper- or lower-body exercise. At a given submaximal BLa and RPE, $\dot{\text{V}}$O_2_ and PO were higher in lower- compared with upper-body exercise, both when expressed as absolute values or as % of the modality-specific peak (Mittelstadt et al., 1995; Calbet et al., 2005). Furthermore, when exercising in a given modality at the same submaximal BLa or RPE, absolute $\dot{\text{V}}$O_2_ and BLa were higher in male compared with female athletes, while sex-differences disappeared when expressed as % of modality-specific $\dot{\text{V}}$O_2peak_ and PO_peak_ (Robertson et al., 2000; Larsson et al., 2002; Garcin et al., 2005; Hegge et al., 2016). Limited evidence suggests that, at a given submaximal BLa and RPE, Δ_UP−RUN_ in absolute $\dot{\text{V}}$O_2_ and PO was larger in female compared with male XC skiers (unpublished submaximal data of Hegge et al., 2015). Assuming altered training in recent years, we expect that the Δ_UP−RUN_ differences between male and female athletes have been reduced or even closed also at submaximal intensity.

This study aimed to investigate the interaction between exercise modality [i.e., upper-body poling (UP) and running (RUN)] and sex (i.e., female and male XC skiers) in physiological responses and power output (PO) across the entire intensity spectrum (i.e., from low to maximal intensity).

## Methods

### Participants

Ten female (age: 23 ± 3 years, body mass: 64 ± 4 kg, height: 171 ± 4 cm, annual training: 702 ± 200 h) and 10 male (age: 23 ± 2, body mass: 80 ± 6, height: 182 ± 3, annual training: 779 ± 120 h), highly endurance trained XC skiers, participated in the study. The inclusion criteria for this study were as follows: participation in the Norwegian championships, World Cup races, or other highly ranked International Ski Federation races in the 2016/17 season. The performance level, based on International Ski Federation's ranking (FIS) points, was similar for the female and male XC skiers (137 ± 64 vs. 142 ± 76 points, respectively). All participants signed a written informed consent form before the data collection and were made aware that they could withdraw from the study without giving a reason. The study was approved by The Norwegian Center for Research Data (ID 51389) and conducted in accordance with the Declaration of Helsinki.

### Overall Study Design

The participants performed stepwise incremental tests with 5-min stages from low intensity to exhaustion during RUN on the first day and UP on the second day. Brief breaks between stages allowed for the collection of a blood sample for the determination of BLa. The participants were instructed to refrain from food intake 2 h before, caffeine intake the day of, and heavy training and alcohol 24 h before testing (American College of Sports Medicine, 2013).

### Test Protocol

**Figure 1:**
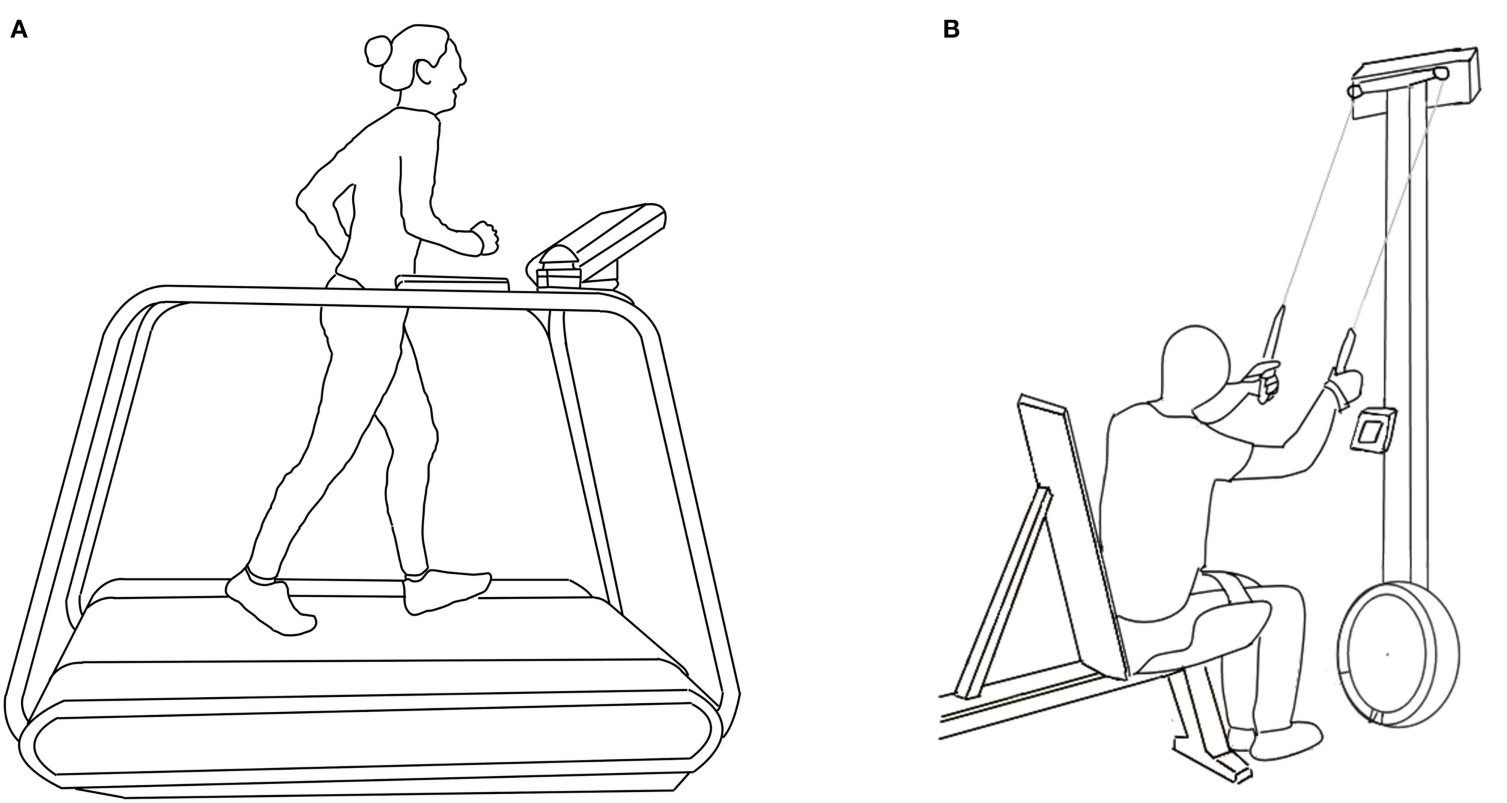
Test setup for running (RUN; **A**) and upper-body poling (UP; **B**) on a Concept2 ski ergometer while being strapped into a modified weight-lifting bench.

Prior to the incremental test for RUN, the participants performed a 10-min warm-up at an intensity of 60–70% of their self-reported HR_peak_. Based on the individual speed during the warm-up, the first 5-min stage of the incremental test during RUN started at 8.5 or 9.5 km·h^−1^ for the male and 7.5 or 8.5 km·h^−1^ for female XC skiers. Thereafter, the workload increased by 1 km·h^−1^ for 5–7 5-min stages until the participants reached exhaustion or if a further increase in speed was not possible. The RUN warm-up and incremental test were performed at a 10% incline on a Woodway treadmill (Woodway GmbH, Weil am Rhein, Germany) (Figure 1A). The 10% incline was chosen to avoid that the inability to maintain high speeds at a flat incline limits the physiological responses (Paavolainen et al., 2000; De Lucas et al., 2021). RUN was chosen as test modality as opposed to cycling since the included XC skiers used RUN more frequently during their training.

The 10-min warm-up for UP was performed at a stable PO corresponding to 7–8 in RPE on the 6–20 Borg scale (Borg, 1982). The first stage of the incremental test started at the PO corresponding to an RPE of 8. The PO thereafter increased with 20 W for the male and 15 W for the female XC skiers for 5–9 5-min stages until exhaustion was reached or a further increase in PO was not possible. The UP warm-up and incremental test were performed on a Concept2 ski ergometer (Concept2 Inc., Morrisville, VT, USA), which is a commonly used modality to investigate isolated upper-body endurance performance in XC skiers (Hegge et al., 2015; Baumgart et al., 2017; Undebakke et al., 2019). The participants sat on a modified weightlifting bench while being tightly strapped around the pelvis and thighs to minimize the power contribution from the lower extremities (Figure 1B). The backrest of the bench was positioned at a ≈120° angle to avoid contact with the participants' back during the test, and the participants maintained a ≈90° angle at the knees. The distance from the bench to the ski ergometer was set according to individual preference. The participants used XC pole straps to tighten the hands firmly to the ergometer handles during UP.

$\dot{\text{V}}$O_2_ was measured by open-circuit indirect calorimetry using an Oxycon Pro ergospirometer (Jaeger GmbH, Hochberg, Germany) during the last 2–3 min of each stage and the entire final stage during RUN, and throughout the entire stages during UP. Prior to testing, the gas analyzer of the ergospirometer was calibrated against a known mixture of gases (15% O_2_ and 5% CO_2_) and ambient air. The calibration of the flow transducer was manually performed with a 3 L high precision syringe (Hans Rudoph Inc., Kansas City, MO, USA) before each test. During both exercise modalities, heart rate (HR) was continuously measured throughout the test session using a Polar m400 HR monitor (Polar Electro Oy, Kempele, Finland). PO was recorded by the built-in Concept2 software for UP. The exercise was briefly interrupted (i.e., 1–2 min) after each stage in both modalities, for the collection of a blood sample (20 μl) from the fingertip. This blood sample was then used to analyze BLa with the Biosen C-lin lactate measurement system (EKF Industrial electronics, Magdeburg, Germany). The overall RPE also recorded using the 6–20 Borg scale(Borg, 1982).

### Data Processing and Analysis

Data processing was performed in MATLAB (R2019b; Mathworks Inc., Natick, MA, USA). For the comparison of PO between the exercise modalities, the PO for each stage during RUN was calculated as the power against gravity (Equation 1) (Sandbakk et al., 2014):


(1) $$\begin{array}{l} {\text{P}}_{\text{g}}=\text{m}\cdot \text{g}\cdot \text{sin}\left(\text{α}\right)\cdot \text{v} \end{array}$$

Where *m* is the mass of the participant, *g* is the gravitational acceleration, α is the incline of the treadmill, and *v* is the belt speed. The PO during UP was recorded as 20-s averages. During both modalities, $\dot{\text{V}}$O_2_ was recorded as 10-s averages and HR every s. Steady-state PO, $\dot{\text{V}}$O_2_, and HR were calculated as averages over the last minute of each stage and the highest values were defined as PO_peak_, $\dot{\text{V}}$O_2peak_, and HR_peak_.

For each participant, the independent parameters (BLa and RPE) were regressed against the dependent parameters [absolute and body-mass normalized $\dot{\text{V}}$O_2_, HR and PO, and % of modality-specific $\dot{\text{V}}$O_2peak_ (%$\dot{\text{V}}$O_2peak_), % of modality-specific HR_peak_ (%HR_peak_), and % of modality-specific PO_peak_ (%PO_peak_)]. The dependent parameters were then inter- and extrapolated to cover 1.5–11.1 mmol·L^−1^ BLa and 7–19 RPE. Mixed ANOVA were used to investigate the differences in peak physiological responses, PO_peak_, and RPE between modalities (i.e., RUN and UP; within-subjects factor) and sex (i.e., female and male XC skiers; between-subjects factor), as well as the interaction between modalities and sex. To make the same comparisons across the entire exercise intensity spectrum, statistical parametric mapping (SPM) with mixed-measures ANOVA were performed in MATLAB by employing the SPM toolbox (github.com/0todd0000/spm1dmatlab) (Pataky et al., 2016). An α level of 0.05 was used to indicate statistical significance.

## Results

### Peak Physiological Responses and Perceived Exertion

**Figure 2:**
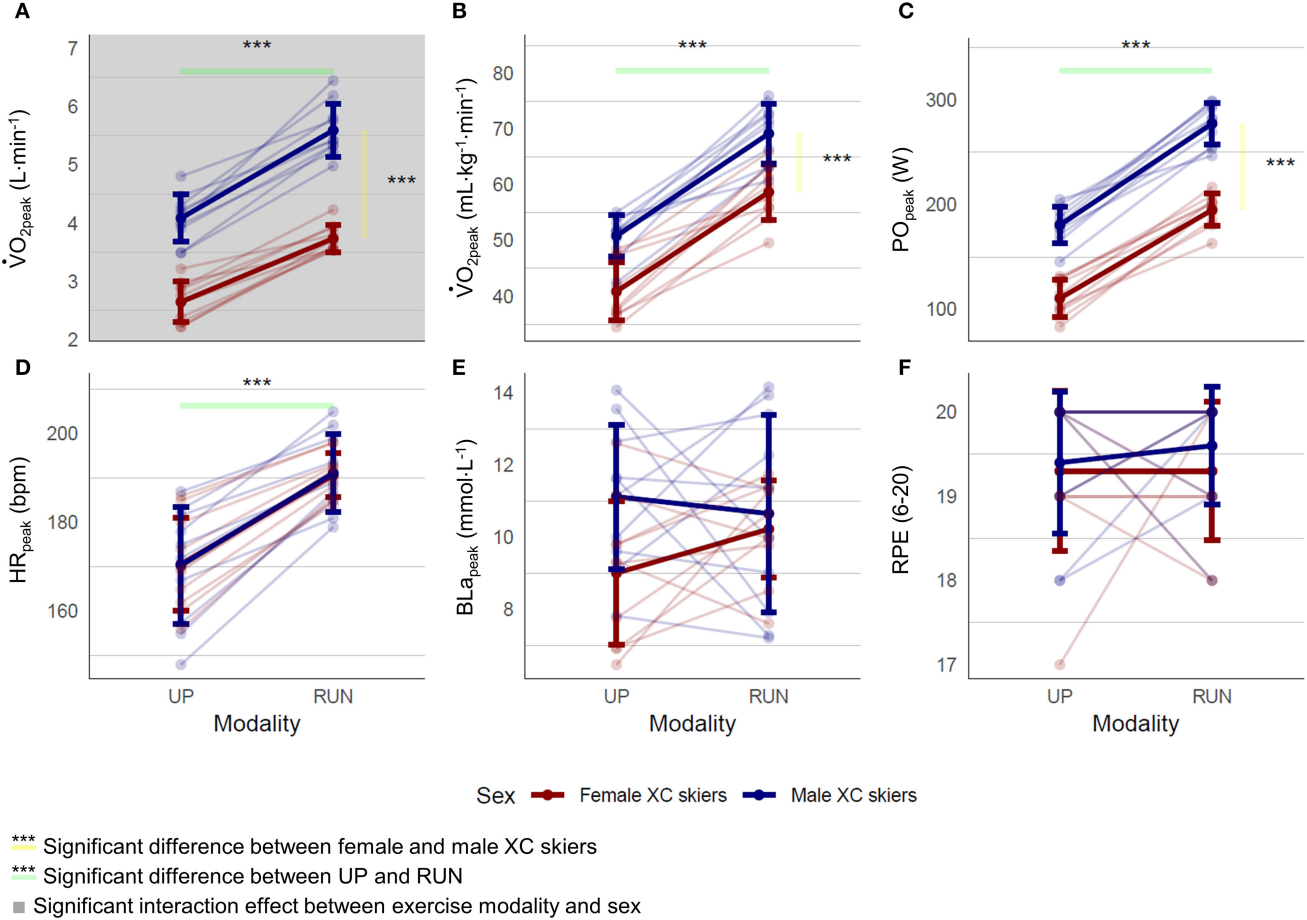
Peak physiological **(A, B, D, E)** and perceputal responses **(F)**, as well as peak power output **(C)** during an incremental running (RUN), and upper-body poling (UP) test to exhaustion in 10 female and 10 male highly trained XC skiers. $\dot{\text{V}}$O_2peak_, Peak oxygen uptake; PO_peak_, peak power output; HR_peak_, peak heart rate; BLa_peak_, peak blood lactate; RPE, peak rate of perceived exertion.

An overview of the peak values obtained during the incremental tests is presented in Figure 2. Absolute and body-mass normalized $\dot{\text{V}}$O_2peak_, as well as PO_peak_, were significantly lower in UP compared to RUN and significantly lower in the female compared with the male skiers. There were no significant interaction effects between exercise modality and sex for most of the investigated responses (*p* > 0.197), i.e., Δ_UP−RUN_ did not differ between female and male XC skiers. The only exemption to this was absolute $\dot{\text{V}}$O_2peak_ with larger Δ_UP−RUN_ differences in the male compared with the female XC skiers (1.5 ± 0.5 vs. 1.1 ± 0.3 L·min^−1^, respectively, *p* = 0.03), although differences were similar when expressed relatively as the ratio of UP·RUN^−1^ (i.e., 73% for the male and 72% for the female XC skiers).

### Physiological Responses and Perceived Exertion Across the Entire Exercise Intensity Spectrum

**Figure 3:**
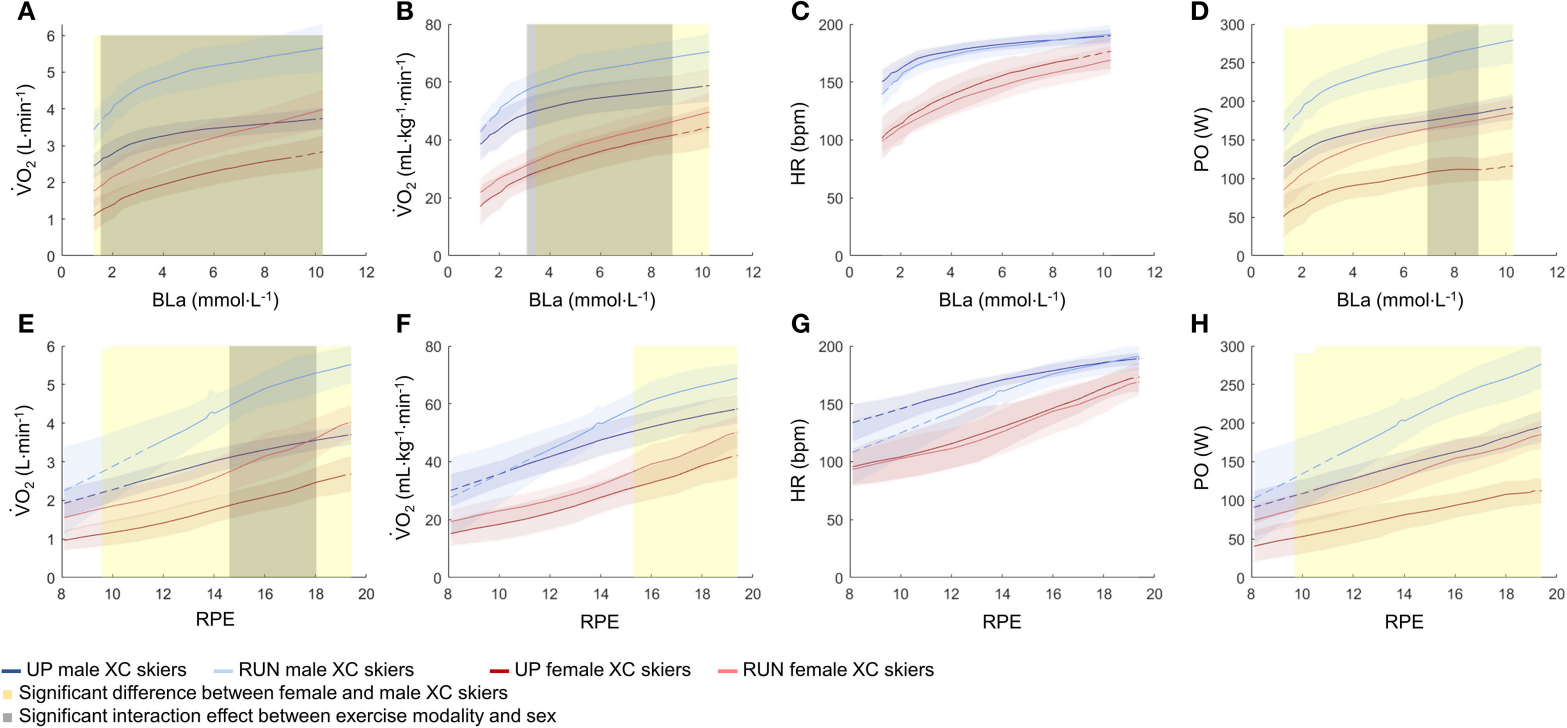
$\dot{\text{V}}$O_2_ **(A,B,E,F)**, HR **(C,G)**, and PO **(D,H)** plotted against BLa and RPE across the entire exercise intensity spectrum from submaximal to maximal intensity during running (RUN) and upper-body poling (UP) for the highly trained female (*n* = 10) and male (*n* = 10) XC skiers. All values are presented as mean ± SD. Based on the statistical parametric mapping, the shaded areas indicate statistical significance at an α level of 0.05. Note that the shaded areas indicating significantly lower values for all parameters during upper-body poling compared to running across the entire exercise intensity spectrum are not displayed. The dotted lines mark the areas where data were extrapolated. $\dot{\text{V}}$O_2_, oxygen uptake; HR, heart rate; PO, power output; BLa, blood lactate concentration; RPE, rating of perceived exertion.

At a given BLa and RPE, absolute and body-mass normalized $\dot{\text{V}}$O_2_, as well as PO, were lower in UP compared to RUN across the entire exercise intensity spectrum, and lower in the female compared with male XC skiers across most of the exercise intensity spectrum (Figure 3). Notably, the significant interaction effects between exercise modalities and sex for absolute and body-mass normalized $\dot{\text{V}}$O_2_ as well as PO, occurred due to larger differences between UP and RUN in male compared with female XC skiers.

**Figure 4:**
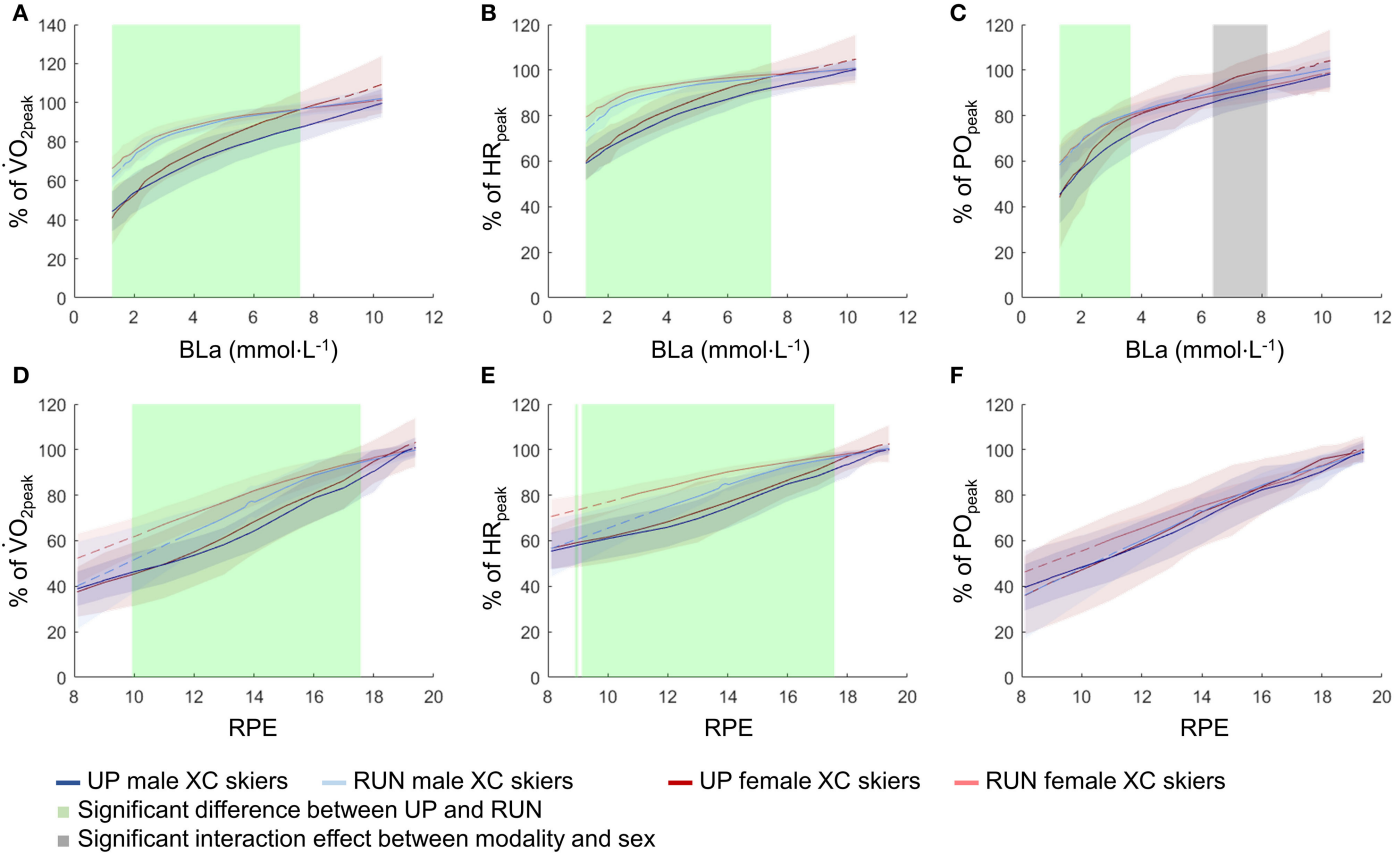
$\dot{\text{V}}$O_2peak_ **(A,D)**, HR_peak_ **(B,E)**, and PO_peak_ **(C,F)** as % of the respective modality-specific peak value plotted against BLa and RPE across the entire exercise intensity spectrum from submaximal to maximal intensity during running (RUN) and upper-body poling (UP) both for highly trained female (*n* = 10) and for male (*n* = 10) XC skiers. All values are presented as mean ± SD. Based on the statistical parametric mapping, the shaded areas indicate statistical significance at an α level of 0.05. The dotted lines mark the areas where data were extrapolated. %$\dot{\text{V}}$O_2peak_, % of modality-specific peak oxygen uptake; %HR_peak_, % of modality-specific peak heart rate; %PO_peak_, % of modality-specific peak power output; BLa, blood lactate concentration; RPE, rating of perceived exertion.

At a given BLa and RPE, the relative physiological responses were higher in RUN compared with UP (Figure 4). Other than the one shaded grey area in Figure 4C, there was no significant interaction effect between exercise modality and sex.

## Discussion

This study investigated the interaction between exercise modality (i.e., UP and RUN) and sex in physiological responses and power output (PO) across the entire intensity spectrum (i.e., from low to maximal intensity). In line with our hypothesis, no significant interaction effects between exercise modalities and sex were found for any investigated peak parameters other than absolute $\dot{\text{V}}$O_2peak_. Although Δ_UP−RUN_ in absolute and body-mass normalized $\dot{\text{V}}$O_2_ at a given submaximal BLa and RPE were larger in the male compared with female XC skiers, these interactions disappeared when expressed as % of the modality-specific peak.

Except for absolute $\dot{\text{V}}$O_2peak_, we did not find interaction effects for any of the investigated peak parameters, i.e., there were no sex differences in Δ_UP−RUN._ This is in contrast to previous research, where Δ_UP−RUN_ differences in $\dot{\text{V}}$O_2peak_ and PO_peak_ were larger in female skiers (Sandbakk et al., 2012; Hegge et al., 2016). A possible explanation of the current findings may be that many female skiers now incorporate more upper-body specific endurance and strength training than before, and have thereby closed the gap in Δ_UP−RUN_ to their male counterparts. In contrast, the only peak parameter with an interaction effect was absolute $\dot{\text{V}}$O_2peak_ with larger Δ_UP−RUN_ differences in the male skiers. These larger Δ_UP−RUN_ differences in absolute $\dot{\text{V}}$O_2peak_ can solely be attributed to the larger magnitude of this parameter in male skiers for both modalities and disappeared when expressed as a ratio UP·RUN^−1^ with average values of 73% for the male and 72% for the female XC skiers. In this context it should be mentioned that inherent exercise modality and sex differences remain, and that peak parameters were generally lower in UP compared with RUN, and in the female compared with male XC skiers. In brief, the Δ_UP−RUN_ differences are attributed to smaller muscle mass, earlier recruitment of fast-twitch muscle fibers, lower aerobic capacity, and higher catecholamine levels during upper-body compared with lower-body exercise (Sawka et al., 1982; Rusko, 1989; Van Hall et al., 2003). The lower $\dot{\text{V}}$O_2peak_ and PO_peak_ in female compared with male athletes within modalities are attributed to lower skeletal-muscle mass, higher body fat, smaller size of O_2_ transport organs (i.e., the heart), and lower levels of hemoglobin (Cureton et al., 1986; Miller et al., 1993; Janssen et al., 2000).

At submaximal intensity, interactions were found for absolute and body-mass normalized $\dot{\text{V}}$O_2_ and PO, with larger Δ_UP−RUN_ differences for the male compared with female the skiers. This was caused by higher $\dot{\text{V}}$O_2_ and PO in the male skiers especially during RUN, which is in contrast to our hypothesis of female skiers having reduced the Δ_UP−RUN_ differences. However, the interactions between exercise modality and sex for submaximal $\dot{\text{V}}$O_2_ and PO along with sex differences disappeared when being expressed as a percentage of the modality-specific peak. The absence of interactions indicates that female skiers have closed the gap to male skiers also at submaximal intensity. In line with previous studies (Robertson et al., 2000; Larsson et al., 2002; Garcin et al., 2005; Hegge et al., 2016), the lack of differences in relative parameters for female and male skiers within a specific exercise modality indicates that no sex-related considerations need to be made when regulating endurance training intensity as a percentage of peak HR, $\dot{\text{V}}$O_2_, or PO.

The higher relative physiological responses in RUN compared with UP at any given submaximal BLa and RPE for both sexes are mostly supported by earlier studies. In line with the current study, Mittelstadt et al. (1995) and Calbet et al. (2005) found a higher %$\dot{\text{V}}$O_2peak_ at given BLa values in whole-/lower-body compared with upper-body exercise. Apparently, during RUN, where greater muscle mass is employed, exercise can be sustained at higher relative physiological values (i.e., %$\dot{\text{V}}$O_2peak_ and %HR_peak_). In contrast, Undebakke et al. (2019) did not find differences in %HR_peak_ at a given RPE between the upper- and whole-body exercise. Thus, their conclusion that %HR_peak_ and RPE can be used interchangeably between RUN and UP for intensity regulation (Undebakke et al., 2019) should be further examined before a final conclusion can be drawn.

### Methodological Considerations

To investigate the interactions between exercise modality and sex in external load, we calculated PO for RUN based on a power balance model, while PO calculations for UP were based on the Concept2 skiergometer's internal software, which uses a model of rotational inertia. However, there are some limitations to these approaches. For RUN, PO is estimated only from the elevation of the center of gravity, neglecting the horizontal component of propulsion (Kram and Taylor, 1990), thereby slightly underestimating PO. For UP, on a similar Concept2 rowing ergometer, PO was shown to be underestimated by about 25 W (Boyas et al., 2006). Furthermore, the baseline metabolic cost in UP (performing the movement at 0 W) is relatively low, while in RUN, and this cost is considerably larger (as in running on the flat) (Kram and Taylor, 1990). As such a considerable metabolic cost occurs at zero PO during RUN, but not during UP. While these limitations may affect the magnitude of the absolute difference between UP and RUN, they do not affect female and male athletes differently. Given that we were mainly interested in whether Δ_UP−RUN_ differed between female and male athletes, these limitations are unlikely to affect the general outcome of this study.

Furthermore, an intermittent protocol was used to allow measurements of BLa between stages. This intermittent protocol led to gaps in the gas exchange data; gaps which we filled by interpolation. While this may have led to minor inaccuracies, we do not think that it affected the overall patterns presented in this study. In addition, participants started their incremental tests at a similar BLa during RUN and UP, whereas the corresponding starting RPE was higher in RUN than UP. To compare physiological responses between RUN and UP both at lower perceived intensities, we extrapolated these at RPE values from ≈7–12 for RUN. Therefore, the extrapolated ranges, which are indicated by the dotted lines in Figures 3, 4 should be interpreted with caution.

## Conclusion

No significant interaction effects between exercise modality (i.e., UP and RUN) and sex were found for the majority of investigated peak parameters. While peak responses were lower for female XC skiers in both modalities, the differences between UP and RUN were not larger in female XC skiers. This indicates that a larger focus on upper-body strength and endurance training in recent years in female XC skiers may have closed the gap between upper-body and running exercise performance as compared to male XC skiers. In line with this, relative physiological responses at a given BLa or RPE did not differ between female and male XC skiers, and no considerations need to be taken when using these values for intensity regulation within a specific exercise modality.

## Data Availability Statement

The original contributions presented in the study are included in the article/Supplementary Materials, further inquiries can be directed to the corresponding author.

## Ethics Statement

The studies involving human participants were reviewed and approved by Norwegian Center for Research Data. The participants provided their written informed consent to participate in this study.

## Author Contributions

LH, ØS, GE, and JKB: conceptualization and methodology. LH and JKB: formal analysis and investigation and writing—original draft preparation. ØS and GE: writing—review and editing and funding acquisition. All authors contributed to the article and approved the final version.

## Funding

This study was funded by the Centre for Elite Sports Research, Department of Neuromedicine and Movement Sciences, Norwegian University of Science and Technology. The funder had no role in the study design; how the data analyses were performed, the decision to publish, or the preparation of the manuscript.

## Conflict of Interest

The authors declare that the research was conducted in the absence of any commercial or financial relationships that could be construed as a potential conflict of interest.

## Publisher's Note

All claims expressed in this article are solely those of the authors and do not necessarily represent those of their affiliated organizations, or those of the publisher, the editors and the reviewers. Any product that may be evaluated in this article, or claim that may be made by its manufacturer, is not guaranteed or endorsed by the publisher.
